# Integrating AI into radiology workflow: levels of research, production, and feedback maturity

**DOI:** 10.1117/1.JMI.7.1.016502

**Published:** 2020-02-11

**Authors:** Engin Dikici, Matthew Bigelow, Luciano M. Prevedello, Richard D. White, Barbaros S. Erdal

**Affiliations:** The Ohio State University College of Medicine, Laboratory for Augmented Intelligence in Imaging, Department of Radiology, Columbus, Ohio, United States

**Keywords:** digital imaging and communications in medicine, picture archiving and communication system, AI-based image analysis, radiology workflow

## Abstract

We present a roadmap for integrating artificial intelligence (AI)-based image analysis algorithms into existing radiology workflows such that (1) radiologists can significantly benefit from enhanced automation in various imaging tasks due to AI, and (2) radiologists’ feedback is utilized to further improve the AI application. This is achieved by establishing three maturity levels where (1) research enables the visualization of AI-based results/annotations by radiologists without generating new patient records; (2) production allows the AI-based system to generate results stored in an institution’s picture-archiving and communication system; and (3) feedback equips radiologists with tools for editing the AI inference results for periodic retraining of the deployed AI systems, thereby allowing continuous organic improvement of AI-based radiology-workflow solutions. A case study (i.e., detection of brain metastases with T1-weighted contrast-enhanced three-dimensional MRI) illustrates the deployment details of a particular AI-based application according to the aforementioned maturity levels. It is shown that the given AI application significantly improves with feedback coming from radiologists; the number of incorrectly detected brain metastases (false positives) decreases from 14.2 to 9.12 per patient with the number of subsequently annotated datasets increasing from 93 to 217 as a result of radiologist adjudication.

## 1 Introduction

Artificial intelligence (AI) (decision trees, regression algorithms, support vector machines, Bayesian methods, neural networks, etc.) has been utilized for decades to address a variety of medical imaging problems, such as image segmentation^1^ (i.e., finding the borders of a target object), registration^2^ (i.e., visually aligning anatomical parts in single- or multimodality images), detection (i.e., detecting formations/structures), and classification^3^ (i.e., grouping of medical information in subgroups). It can also facilitate information feeds in radiology workflows^4^^,^^5^ (e.g., natural language processing in dictation systems). “Machine learning” (ML) is an application of AI in which the computer (machine) is given data access, and models are used for extracting relevant information from the data. The recent usage of neural networks, which is a well-established ML approach, has gained significant momentum with generalization of the techniques to deeper network architectures, referred to as deep neural networks (DNNs); the complete concept is known as “deep learning” (DL).^6^ Research on deeper architectures shows that the accuracy of the deployed models depends heavily on the amount of relevant information; therefore, access to both past data and the ongoing inflow of new information is critical. Accordingly, DL-based solutions are commonly built on vast amounts of data.^7^

Medical imaging presents multiple challenges for researchers attempting to adopt DL. They include the following: (1) circulation of data between institutions, or even between departments within the same institution, is complicated by various legal barriers largely related to privacy issues; (2) high-resolution and high-dimensionality (e.g., 3-D + time) of the data commonly translates into AI models with high magnitudes of parameters; large amounts of data are then needed for convergence of models; and (3) most medical-imaging applications require image annotations (i.e., segmentation and detection results) by medical experts to train the AI algorithms;^8^^,^^9^ medical images without relevant and accessible annotations might not be useful for a variety of supervised learning scenarios in which the machine learns how to map an input image to output results by processing example input–output pairs. Thus, researchers pursuing the latest developments in ML must restructure their workflows to enable a flow of high-quality annotated information to both train and continuously update medical-imaging models.

Accordingly, this report introduces architectural modifications to a given radiology workflow in multiple stages, delineating research, production, and feedback maturity levels. The ultimate goal of this work is to promote the integration of imaging AI into the radiology workflow in which inference-generating models grow organically with the continuous inflow of both new medical data and radiologist feedback for ongoing model learning. Therefore, the feedback maturity level is the final goal, with research and production serving as prior stages to achieving this end. To further solidify the understanding of these maturity levels, a case study representing the deployment of an example AI application, brain metastases (BM) detection with T1-weighted contrast-enhanced three-dimensional (3-D) MRI, is provided. The results section represents the evaluation of the accuracy of that AI application at three incremental quantities of added feedback data from radiologist adjudication of inference results in a complete simulated deployment [i.e., with 93, 155, and 217 annotated datasets and using a fivefold cross-validation (CV)^10^]. The report concludes with a discussion of the results, system limitations, and future directions.

## 2 Radiology Workflow and Its Adaptations

### 2.1 Example Radiology Workflow and Definitions

**Fig. 1:**
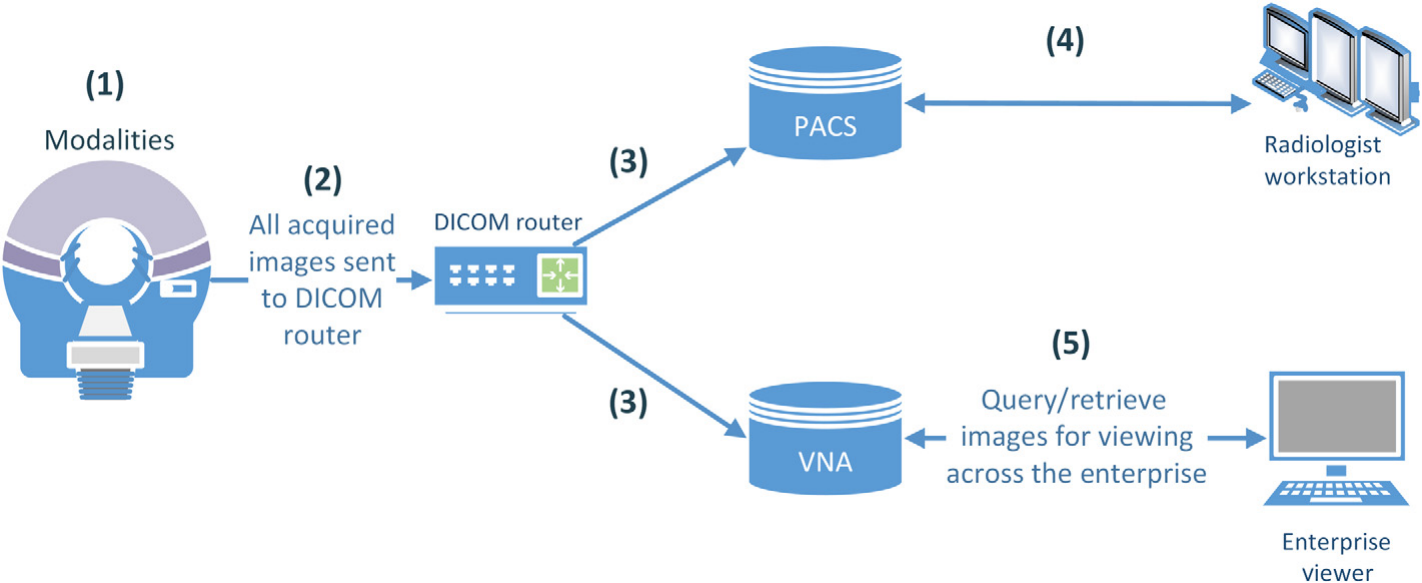
A simplified view of an example radiology workflow.

The implementation and maintenance of radiology workflows have been investigated in numerous earlier studies.^11^^,^^12^ The workflow example highlighted in this study is as follows (see Fig. 1):

(1) Medical images are acquired with a standard modality (e.g., CT, MRI, etc.) by a technologist.
(2) The images acquired in digital imaging and communications in medicine (DICOM) format are sent to a DICOM router,^13^ a configurable framework that is capable of sending/receiving DICOM images to/from predefined addresses.
(3) The router sends the images to the (1) picture archiving and communication system (PACS) and (2) vendor-neutral archive (VNA). The VNA is a technology enabling the storage of medical images in a standard format and offering a generic interface, thereby making the data accessible to multiple healthcare professionals regardless of the type of system from which images are originating.^14^
(4) Using dedicated workstations, radiologists access image data stored in PACS for study visualization, postprocessing, and interpretation.
(5) Nonradiology clinicians may also view medical images stored only in a VNA through links in each patient’s electronic medical record (EMR).

### 2.2 Architectural Adaptations to Achieve Levels of Maturity

#### 2.2.1 Research maturity level

**Fig. 2:**
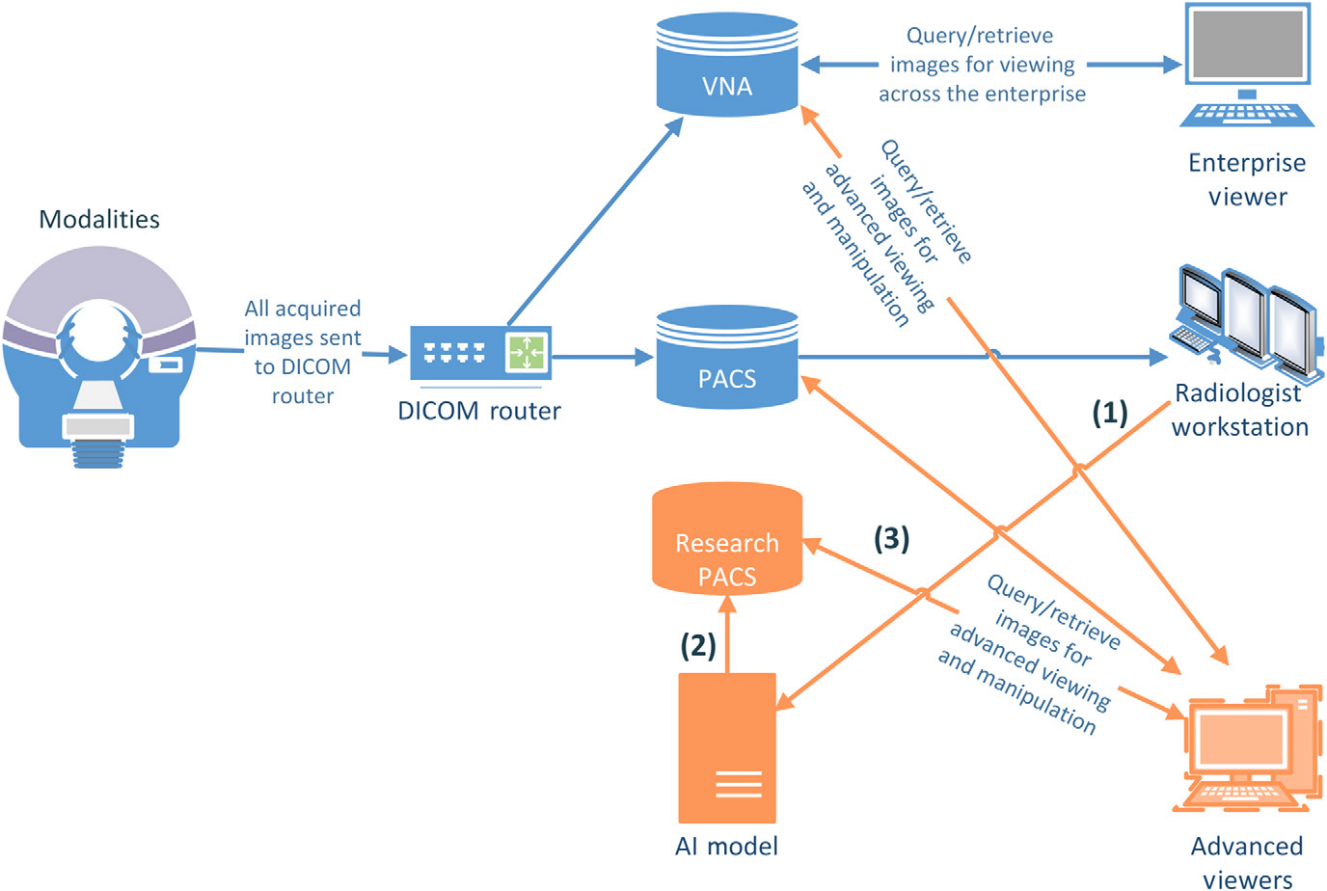
Research workflow.

To represent the inference results from an ML algorithm to a dedicated group of medical experts, the workflow must be adapted as shown in Fig. 2.

At this maturity level, imaging modalities (e.g., CT, MRI, etc.) send acquired images to a DICOM router which then distributes received images to pertinent storage locations, such as the PACS or VNA. The images archived in PACS can then be accessed by a radiologist via dedicated workstations just as the standard workflow. Next, the following occur sequentially:

(1) To benefit from the AI-based algorithm, the radiologist may send images to a DICOM node where the AI system is deployed; the DICOM node can be a virtual machine running an application composed of a DICOM listener and a programmatic component to process the images for inference (e.g., Python script for the AI-based algorithm). The AI system receives the input DICOM images, processes them, and prepares the results as a (1) DICOM mask, where an algorithm’s output is represented as another DICOM image with voxel-values representing the findings; (2) grayscale softcopy presentation state (GSPS) object;^15^ (3) DICOM segmentation (DICOM SEG) object;^16^ or (4) DICOM structured report (DICOM SR).^17^
(2) After the result dataset is generated, it is sent to a separate research-PACS that utilizes both (1) DICOM message service element (DIMSE) and (2) DICOMweb, which specifies a web-based service for accessing and presenting DICOM objects. This allows the official image records in PACS, as well as patient EMRs, to remain intact.
(3) The archives in the research-PACS are accessible by stand-alone advanced DICOM viewers that can connect to a variety of DICOM storage locations. Advanced viewers allow radiologists and other medical experts to view and analyze AI system results related to processed DICOM images; they commonly include visualization tools to view output files (i.e., GSPS, DICOM SEG, etc.) in connection with their corresponding standard DICOM images.

#### 2.2.2 Production maturity level

**Fig. 3:**
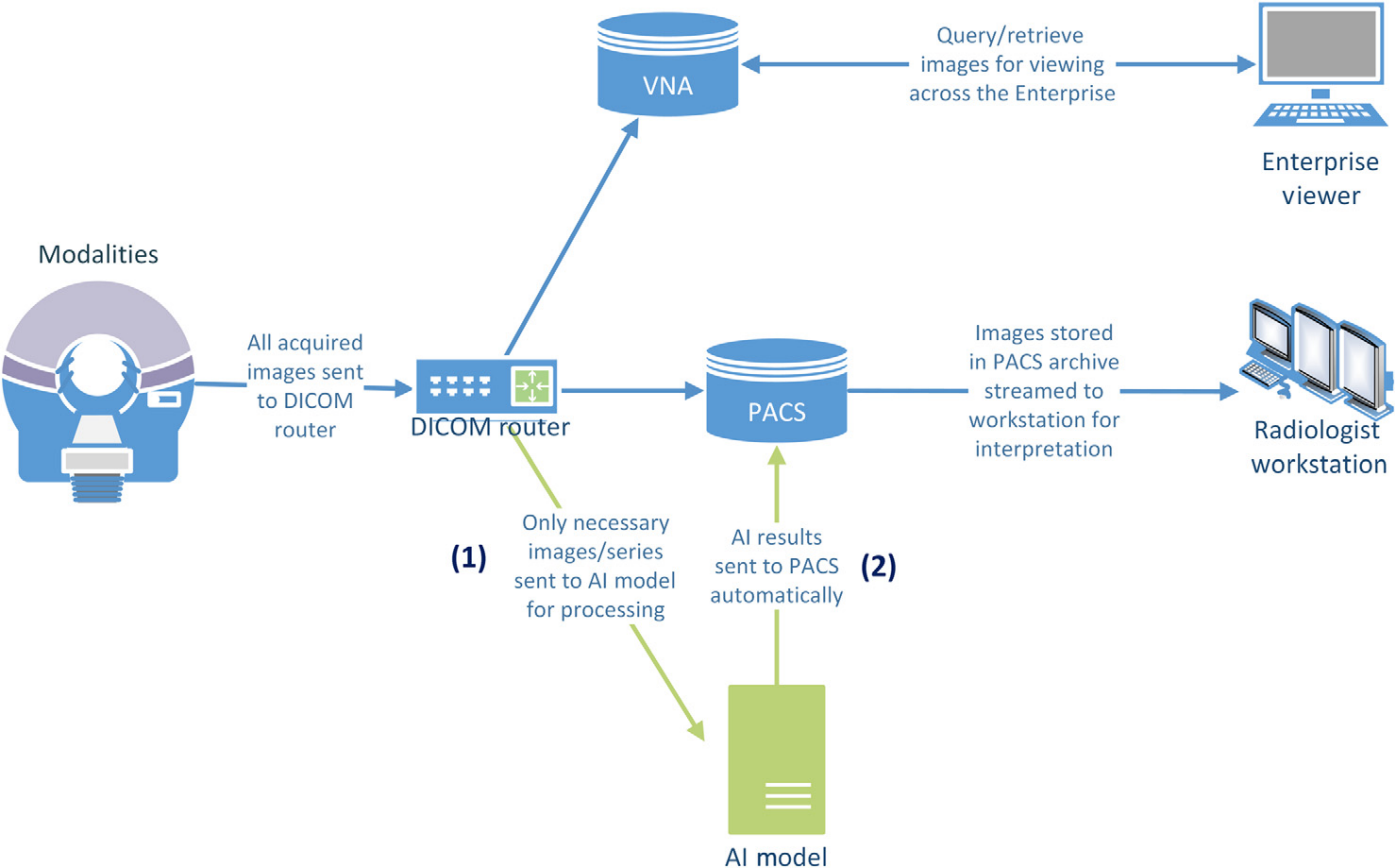
Production workflow.

The production maturity level aims to implement existing AI models as conceived without allowing further modifications. It enables verified AI models that have been deployed, optimized, and validated within the research workflow to be placed in a production mode (see Fig. 3).

At this level of maturity,

(1) An additional path is introduced for acquired images to be sent directly from the DICOM router to the AI system. The received images are processed using a verified model located at the AI system.
(2) AI results are then placed into a PACS system as a DICOM mask, GSPS, DICOM SEG, and/or DICOM SR object. Unlike the research maturity level, the results become part of a patient’s EMR via PACS. Thus, AI results are denoted properly to indicate that they are generated by the AI system.

The production maturity level permits triaging of studies based on the results from the AI model inference; it allows a study to be flagged, or accordingly prioritized, in a radiologist’s reading worklist.^18^ While this setup also allows viewing of AI results in connection with their target images, receipt of radiologist feedback on these results is not facilitated.

Note that the AI results submitted to production PACS have the potential to consume large amounts of archival space on production systems.

#### 2.2.3 Feedback maturity level

**Fig. 4:**
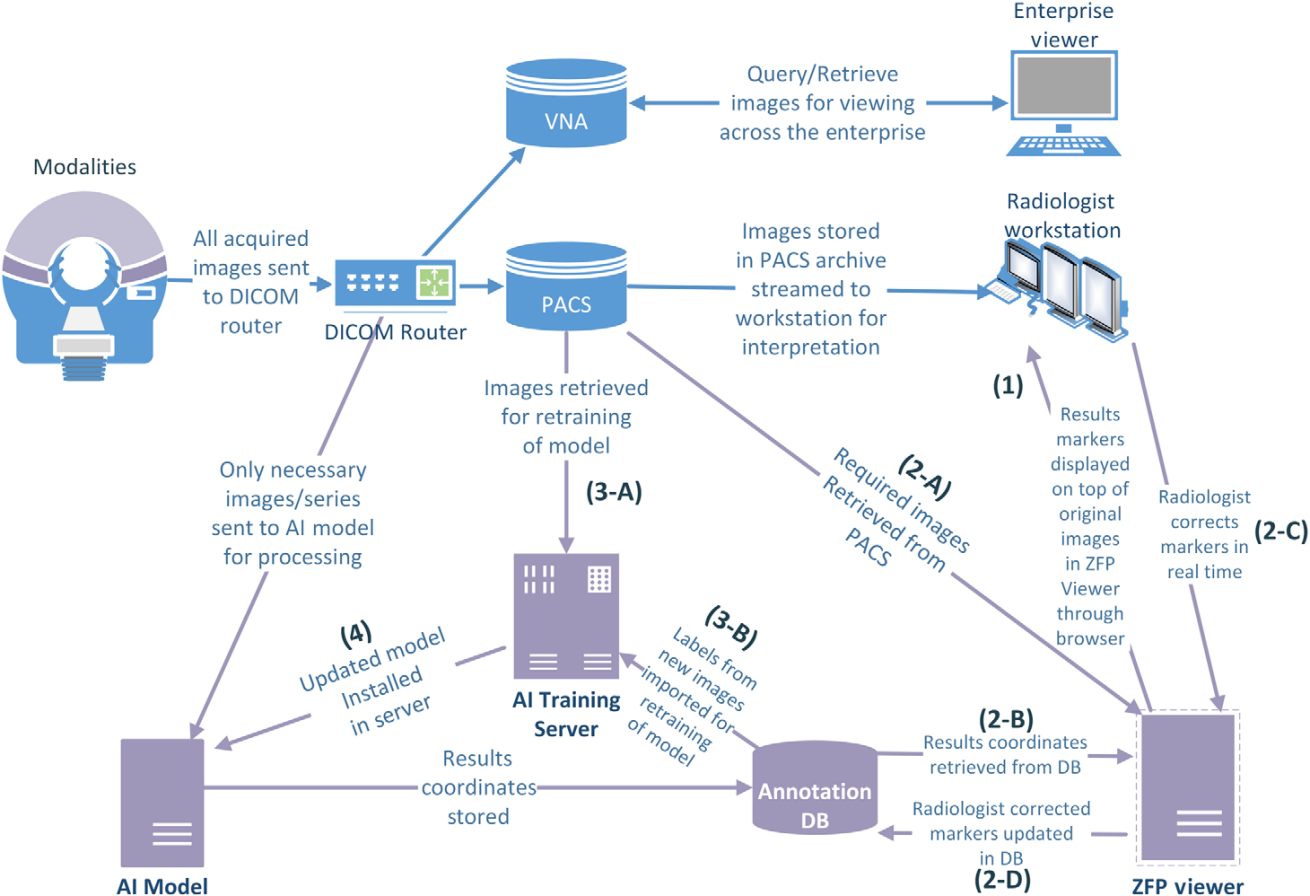
Feedback workflow.

As mentioned earlier, the accuracy of an AI model utilizing DL commonly depends on the amount of data available during the initial training. Accordingly, the feedback maturity level aims to place the AI model at a location where it can benefit from the constant stream of annotated data resulting from radiologist adjudication of inference results; the AI model is continuously updated/modified (see Fig. 4).

The feedback maturity level can be achieved by adding a dedicated AI-model training server, medical-data annotation storage, and medical-imaging viewer that allows adding, editing, and removal of annotations from corresponding medical images. In this workflow,

(1) The medical-imaging viewer can be a web-based zero-footprint (ZFP) application that is triggered by the click of a configurable button in the PACS interface. The ZFP viewer may have single sign-on properties allowing user credentials to be passed automatically; hence, additional login is not required.
(2) The medical-imaging viewer allows (A) visualization of the images by retrieval from PACS, (B) representation of annotations for corresponding images to be stored in a dedicated annotation storage system, and (C) editing/removal of these annotations while (D) storing the modified annotations back to the annotation storage.
(3) The training server is set to periodically access (A) the PACS and (B) annotation server to gather newly acquired data and their annotations for retraining/updating the AI models. The periodicity of retraining can be set depending on a number of factors, such as the data acquisition frequency and workload of the training server.
(4) The updated model is installed as the new AI model.

AI results are tagged with the version of the used AI model, where the version information is linked with the model creation date. The review of the generated AI results is not mandatory, as the results are kept in separate annotation storage. However, if the institution chooses to utilize the full potential of the feedback architecture, then the continuous inflow of radiologist feedback becomes critical.

## 3 Case Study: Brain Metastases Detection Beyond CAD

Computer-aided detection (CAD) technology allows computational procedures to assist radiologists in the diagnosis and characterization of disease by obtaining quantitative measurements from medical images along with clinical information.^19^ A classical CAD system is trained with a collection of medical-imaging datasets before deployment, either as stand-alone software or as a tool in PACS and/or medical imaging viewers. The concept of integrating CAD into PACS (i.e., to allow the execution of CAD procedures on images stored in PACS) has been investigated in previous studies.^20^^,^^21^

The development of a traditional CAD model is complete after the initial training procedure. However, the model may be kept up to date with future batch data updates and training(s), executed as an additional procedure that is not part of a routine radiology workflow.

In this case study, an example AI application for detecting BM with T1-weighted contrast-enhanced 3-D MRI^22^ is deployed in a radiology workflow that evolved through all three aforementioned maturity levels: research, production, and feedback. In the feedback maturity level, this AI application is beyond a traditional CAD; its accuracy is constantly improving due to feedback coming from the interpreting neuroradiologists who receive algorithm inference results while performing clinical interpretations; feedback to the AI system is provided almost seamlessly using the tools that are integrated into the radiology workflow.

### 3.1 Brain Metastases Detection: Research Maturity Level

For the given case study, the technologist acquires the T1-weighted contrast-enhanced 3-D MRI data of a patient using an MRI scanner. The images are sent through DICOM-transfer to a DICOM router. After the images are received by this router, they are forwarded to two different storage locations, including the institution’s PACS and VNA. The images routed to PACS are immediately accessible by neuroradiologists for their interpretation, whereas the images routed to the VNA are accessible to nonradiologist physicians via an enterprise viewer.

**Fig. 5:**
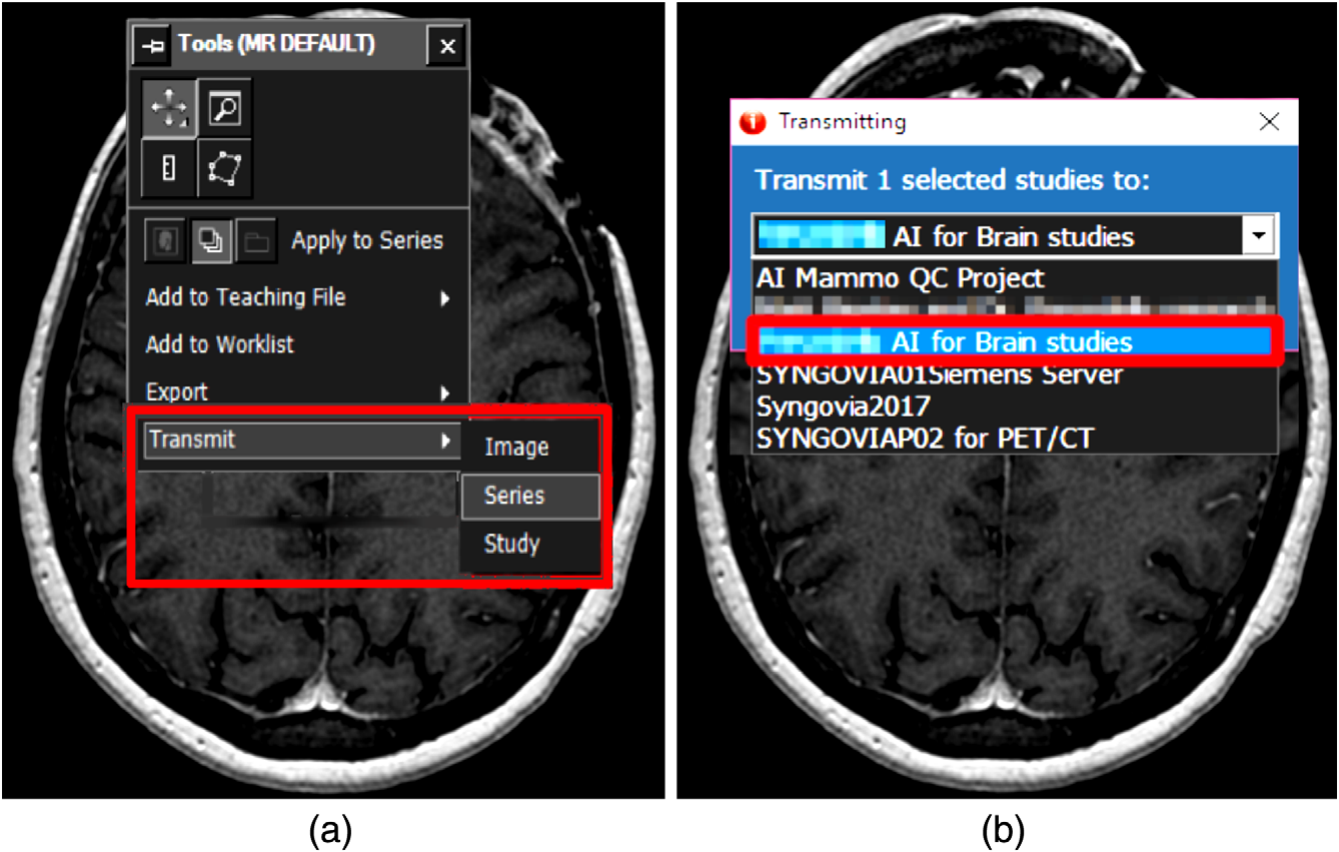
DICOM-transfer to the AI system. (a) DICOM-transfer can be utilized to send the MRI series. (b) The final destination should be the DICOM node where the AI system is located.

During the medical-image interpretation, if the neuroradiologist decides to receive inference results from the AI model, the images can be sent to the AI system via DICOM-transfer, which transmits the series to the DICOM node where the AI system is located (see Fig. 5). This image transfer can be initiated at any time by the neuroradiologist, but it should ideally be done at the beginning of an interpretation to minimize the amount of time waiting on inference results; if images are sent as soon as the examination is opened for interpretation, the neuroradiologist can continue viewing images while the AI model is processing a result.

After processing the 3-D MRI data, the AI model generates a GSPS object to register its results, which are 3-D coordinates of each BM-detected center position in this case. It then uses DICOM-transfer to send the GSPS object to the research-PACS; in the current infrastructure, the results are sent to an advanced imaging-analysis workstation server. In the research architecture, this is a critical phase for keeping the results (1) separated from the patient’s EMR and (2) inaccessible by nonradiology personnel.

**Fig. 6:**
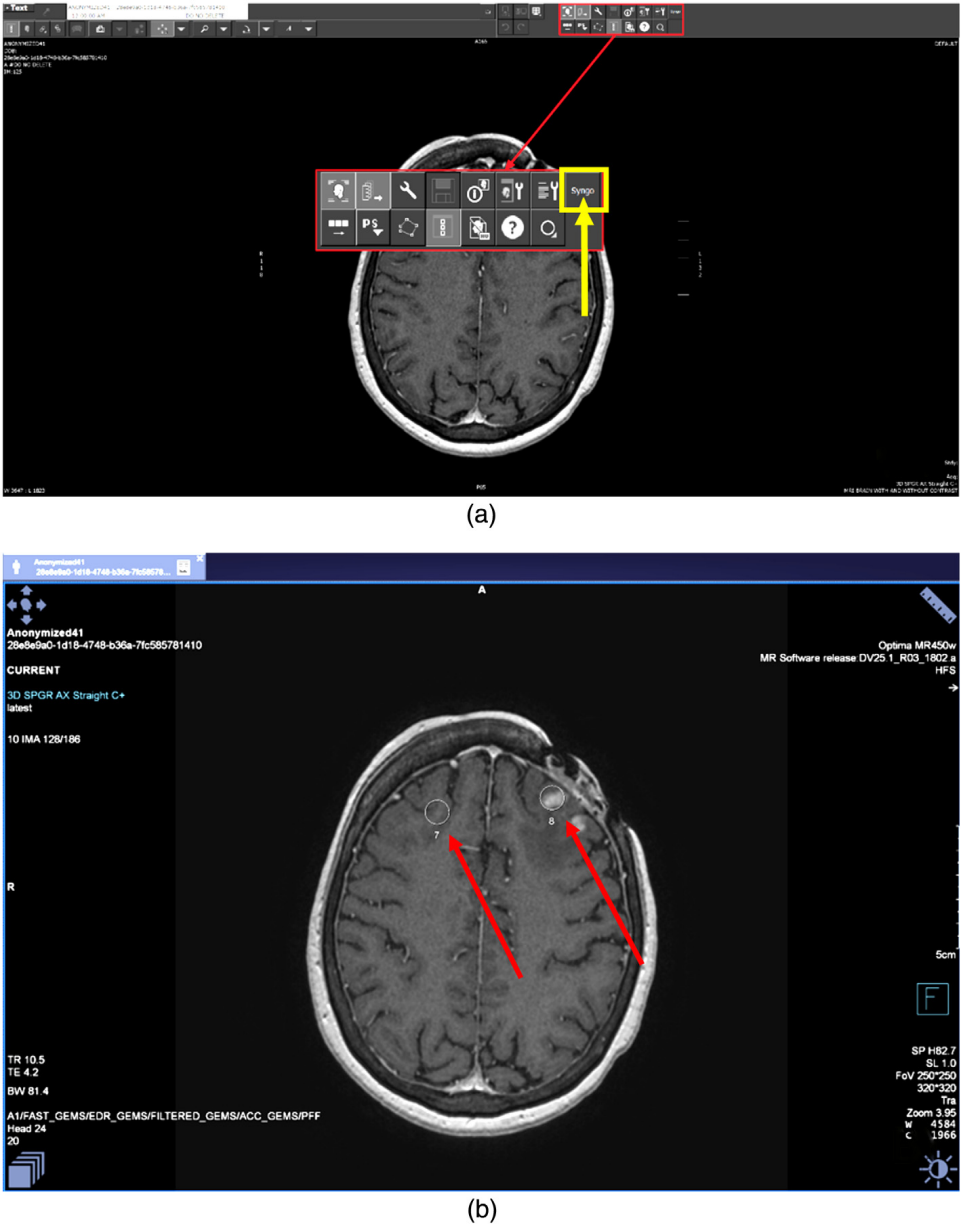
Switching from PACS to the advanced viewer. (a) From the PACS, the neuroradiologist switches to the advanced viewer with the click of a button located at the user toolbar (shown with the yellow arrow). (b) A separate viewer overlays the result, GSPS object, on the medical image (GSPS circle overlays are pointed with red arrows in the figure).

Next, the neuroradiologist can switch to the advanced viewer with the click of a button located in the user toolbar of the PACS. The advanced viewer overlays the resulting GSPS objects on the medical images (see Fig. 6). The visualization of the AI results concludes the capabilities of the research maturity level; the neuroradiologists and medical experts may visually inspect and analyze AI system outputs.

### 3.2 Brain Metastases Detection: Production Maturity Level

After an AI model is approved for clinical usage by the Food and Drug Administration (FDA), the research workflow can be altered slightly to achieve the production maturity level. First, the DICOM router is configured to concurrently send the acquired medical images directly to the institution’s PACS, VNA, and AI systems based on procedure code. The routing rules of the router can be set so that it only forwards the necessary set of images to the AI system; in this case study, the pertinent images are the series for axial T1-weighted 3-D MRI with contrast. For a given patient, the sending of a selected subset of images series, rather than a complete study, is critical; sending a complete study may take significantly more time to transmit while consuming larger storage space at the target destination.

**Fig. 7:**
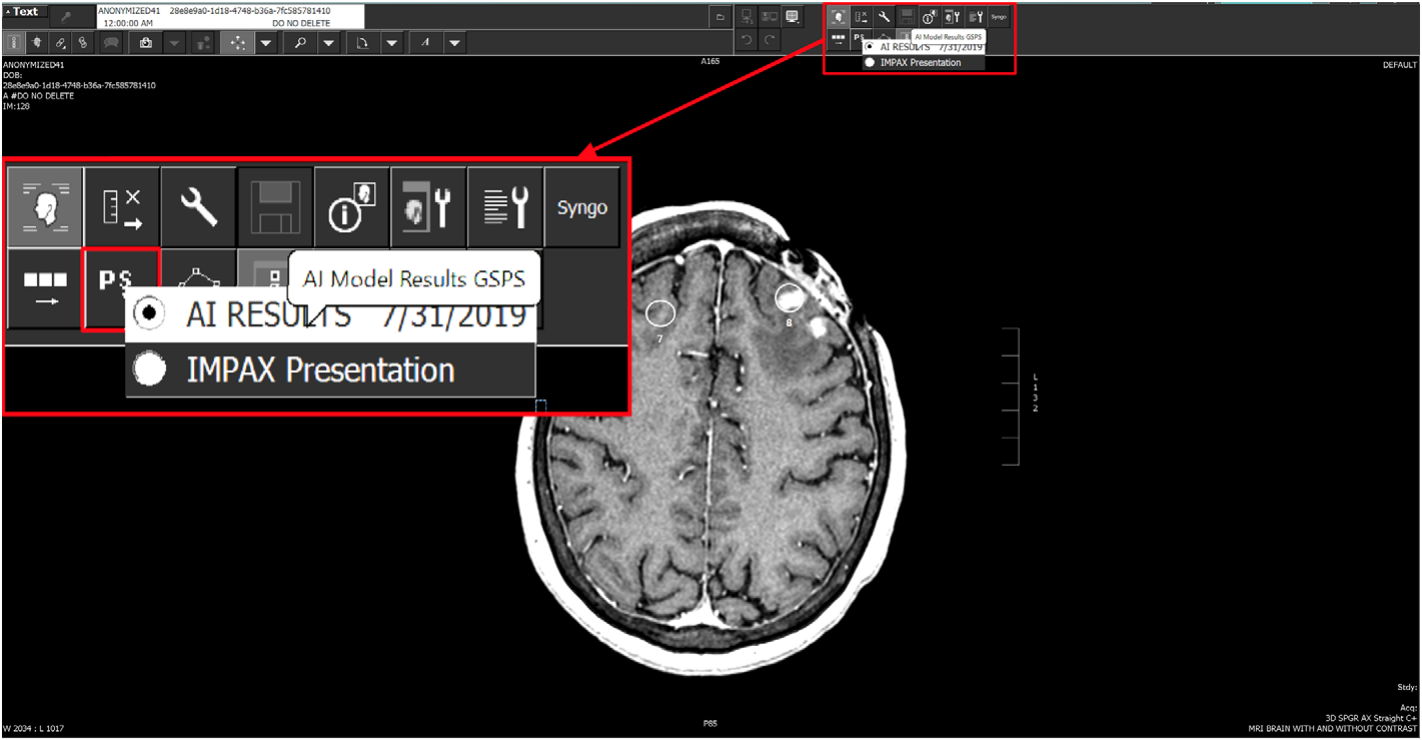
Viewing the AI results from PACS workspace.

After the AI system processes the images received from the router, it sends the results in GSPS format to the PACS server; hence, the results are available for the neuroradiologist to view in their standard PACS workspace. The neuroradiologist can simply load the results by selecting the AI inference presentation state that displays the GSPS overlays on the corresponding MRI data (see Fig. 7).

The production workflow saves the neuroradiologist time by having only the appropriate images automatically routed to the AI model, as well as sending the results to the system where the neuroradiologist will be utilizing them to enhance examination interpretation. The results become part of the patient’s EMR in this architecture; therefore, the deployed AI model must be validated properly during the research architecture deployment.

### 3.3 Brain Metastases Detection: Feedback Maturity Level

**Fig. 8:**
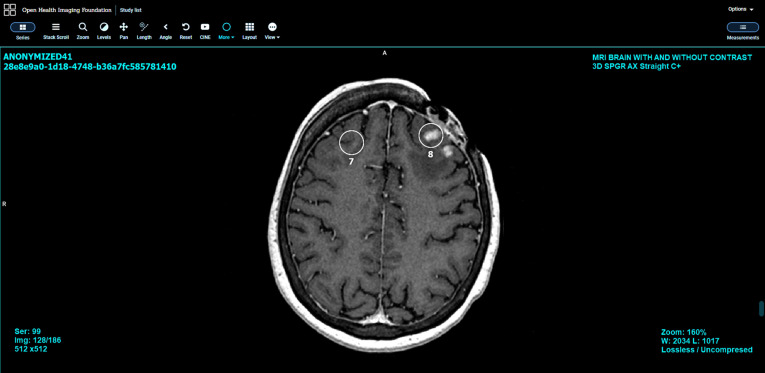
ZFP medical-image viewer showing AI results.

The production architecture can be further modified to integrate feedback processes into the workflow. This is achieved by first incorporating a viewing tool that enables the neuroradiologist to see the AI results/annotations on their corresponding images; the viewing tool must also enable the editing of these annotations. For this purpose, a ZFP medical-image viewer^23^^,^^24^ can be modified for (1) accessing the AI results, representing the AI-detected metastases centers stored in an annotation database and (2) editing/removing these results (see Fig. 8). To enable the continuous updating of the AI model, a dedicated training server can be added into the workflow, where (1) a direct connection between PACS and the training server is established, allowing newly acquired 3-D MRI datasets to be sent from PACS to the training server, and (2) the training server is also given direct access to the annotation database. An updated AI model is automatically used for studies acquired after the model update or for previously acquired studies on demand. By connecting the training server to both PACS and the annotation database, the training server is able to extract labeled data in any desirable format (e.g., GSPS, DICOM SEG, DICOM mask, or DICOM SR).

## 4 Results

The accuracy of the AI model used in the case study^22^ is measured for the feedback maturity level by simulating three incremental quantities of added feedback data from radiologist adjudication of inference results in a simulated complete deployment. The data-selection criteria for these increments were as follows: (1) datasets included 93 (acquired from 85 patients), 155 (from a total of 120 patients, 35 additional patients) and 217 (from a total of 158 patients, 38 additional patients) postgadolinium T1-weighted 3-D MRI exams, respectively.

The major components of this investigation, including (1) the algorithmic details (e.g., DNN architecture, data augmentation steps, training methodology, etc.), (2) analysis of statistical properties of the BM included in the study (e.g., lesion diameter, volume, location, etc.), and (3) adherence to data-acquisition criteria, have been comprehensively described in a previous report.^22^ This retrospective study was conducted under Institutional Review Board approval with waiver of informed consent.

**Fig. 9:**
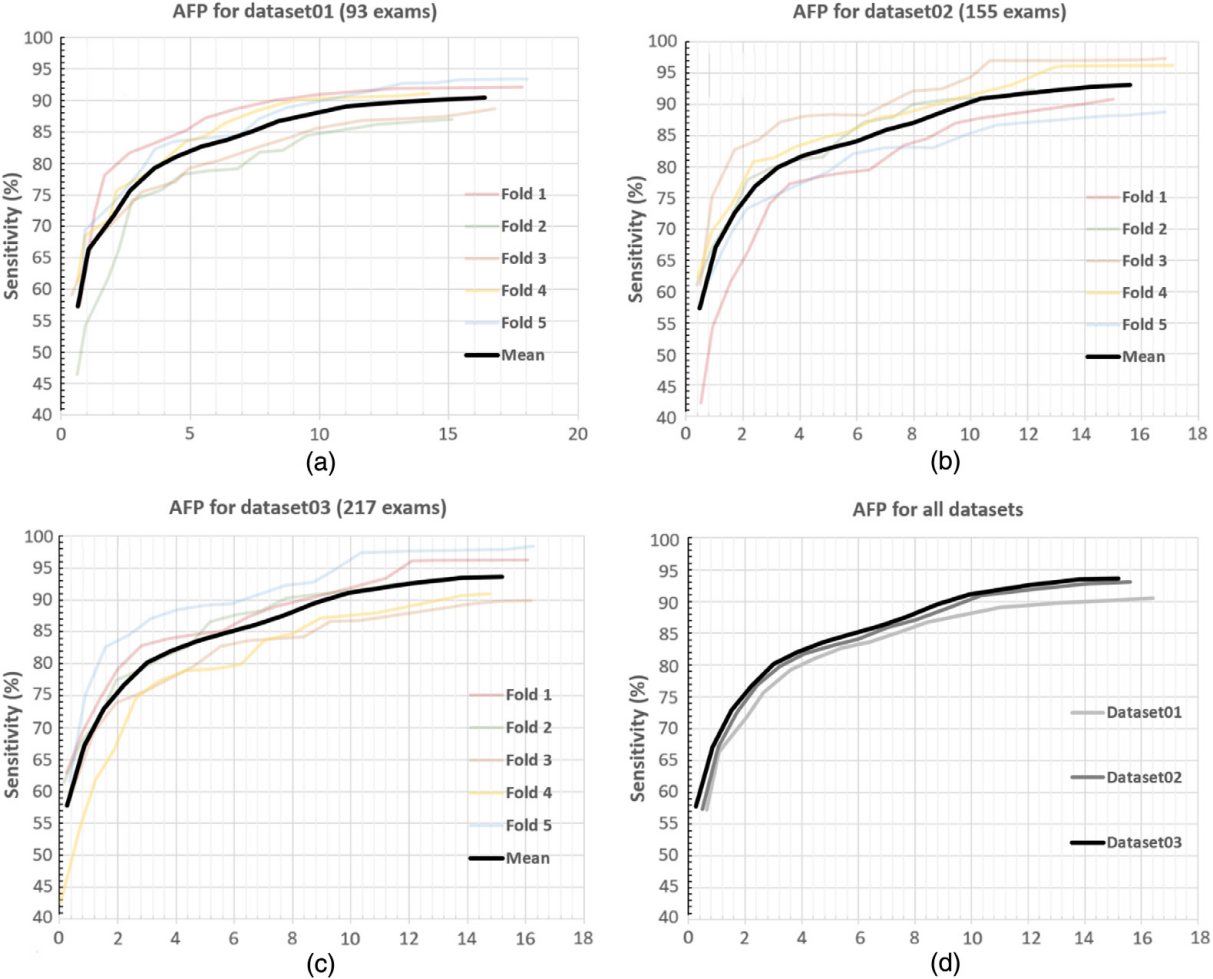
AFP for datasets. Average number of false-positives per patient (i.e., incorrectly detected BM lesions for each patient) in relation to the sensitivity is illustrated for each CV fold and the mean of all folds (black curve) for (a) Dataset01, (b) Dataset02, and (c) Dataset03. (d) The mean AFP curves of all datasets are shown together.

**Fig. 10:**
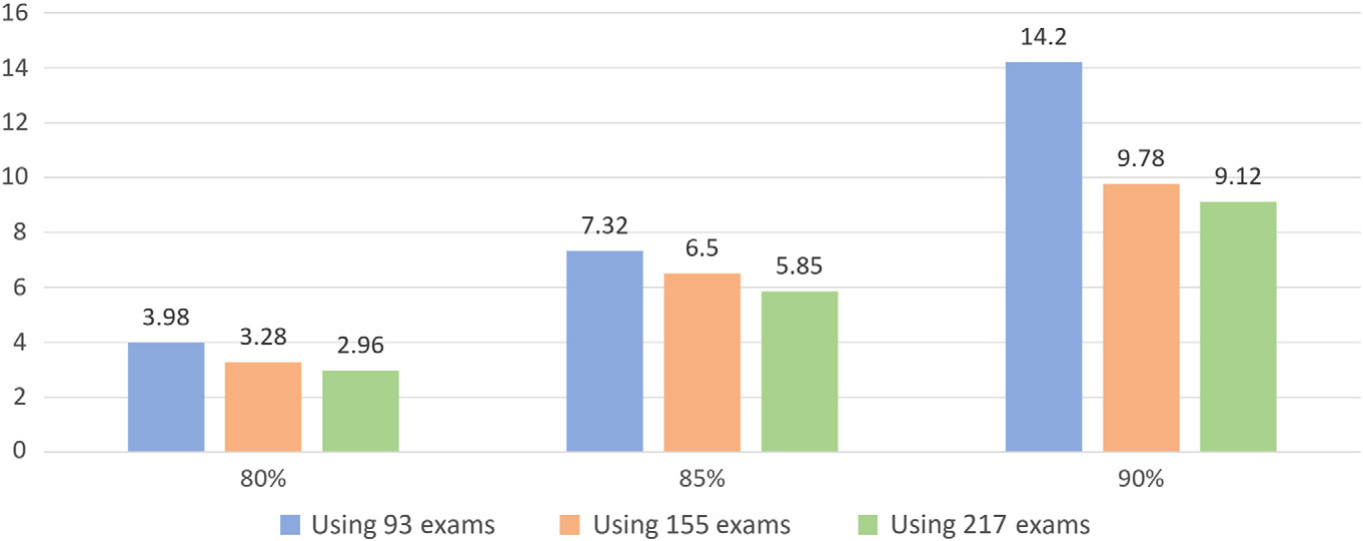
AFP versus sensitivity.

The metric of average false-positives (AFP) per patient, representing the incorrectly AI-detected BM lesions for each patient in relation to the sensitivity, was used during the validation of the algorithm for the three datasets;^21^ the AFP values were computed using a fivefold CV (see Fig. 9). At a 90% sensitivity level (i.e., 90% of true BM are detected for a given test exam), the algorithm produced 14.2, 9.78, and 9.12 false positives per patient for the first [see Fig. 9(a)], second [see Fig. 9(b)], and third [see Fig. 9(c)] datasets, respectively. The reduction of false positives from 14.2 to 9.12 with the addition of 124 exams (i.e., Dataset01 had 93 and Dataset03 had 217 exams) is a significant improvement for a BM detection system. The AFP of the system is summarized for 80%, 85%, and 90% sensitivity levels in Fig. 10.

## 5 Discussion and Conclusion

It has been shown in multiple previous studies that to train more complex models it is commonly better to have more expert annotated data.^25^^,^^26^ The results of this work show that the amount of data is also a determining factor for the accuracy of the AI approach used in the case study. However, the amount of data is not the only element that might benefit an AI-based system; the ML algorithm and its parameters, as well as the properties of the added data (e.g., data labels, image quality, the novelty of the added data, etc.) have a significant impact on the final accuracy. While the feedback maturity level facilitates the gathering of annotated data and feeding of the additional data into deployed models, the selection of proper AI models is still the responsibility of the researchers/data scientists. Accordingly, it would be misleading to assume that the deployment of feedback architecture alone would ensure a successful AI system integration.

The architectures described in this paper should be feasible to implement in most of the medical institutions with relatively modern radiology systems. Variations on institutional setups are common. For example, some institutions may not have a DICOM router between their modalities and PACS. In these cases, the flow of information should be facilitated by other means; either technologists or radiologists could be responsible for transferring the data between systems or more automated processes could be set up within PACS itself (e.g., configured to send data to preset DICOM nodes within the institution). Modern PACS systems are commonly able to load and display GSPS objects; hence, the visualization of basic AI results should not be a limitation. More recent PACS implementations can also accommodate DICOM SEG and/or DICOM SR file types, opening new opportunities for more advanced data display and complex workflows.

As ML enhances its applicability and significance in the medical-imaging domain, radiology workflows enabling AI models to access medical data will become increasingly critical. Accordingly, this report delineates three maturity levels for AI integration into a given radiology workflow: (1) research, representing the results of investigational AI models to radiologists without generating new patient records, (2) production, processing data stored in PACS with a previously validated deployed AI model, and (3) feedback, updating a deployed AI model organically via radiologist interactions with images and their annotations, which allows constant evolution of an AI model from the inference adjudication process. The case study gave implementation directions for these architectures by providing descriptive figures.
